# Efficacy and safety of intensity-modulated radiation therapy versus three-dimensional conformal radiation treatment for patients with gastric cancer: a systematic review and meta-analysis

**DOI:** 10.1186/s13014-019-1294-0

**Published:** 2019-05-22

**Authors:** Fang Ren, Shaodan Li, Yin Zhang, Zhifei Zhao, Haiming Wang, Yixin Cui, Maoyun Wang

**Affiliations:** 1 Department of Traditional Chinese Medicine, Chinese PLA General Hospital, Beijing, 100853 China; 2 Department of Radiotherapy, Chinese PLA General Hospital, Beijing, 100853 China

**Keywords:** Gastric cancer, Intensity-modulated radiation therapy, Meta-analysis, Three-dimensional conformal radiation therapy

## Abstract

**Background:**

Radiation or radiochemotherapy is a common adjuvant therapy for gastric cancer. Intensity-modulated radiation therapy (IMRT) has been demonstrated to provide better dose conformity, allowing dose escalation and/or reduction of normal tissue exposure compared with three-dimensional conformal radiation treatment (3D-CRT). However, the efficacy of IMRT and 3D-CRT in gastric cancer remains controversial. This study aimed to compare the efficacy and safety of IMRT with those of 3D-CRT in treating patients with gastric cancer through conducting a meta-analysis of 3-year survival rates [overall survival (OS) and disease-free survival (DFS)], local control rates, and toxic event rates.

**Methods:**

Embase, PubMed, the Cochrane Library, and clinical trial databases were searched to identify the clinical trials of IMRT versus 3D-CRT for treating patients with gastric cancer. The obtained data of survival and safety were analyzed using the Stata 14.0 software.

**Results:**

A total of 9 controlled clinical studies, including 516 patients with gastric cancer, met the inclusion criteria and were included in this meta-analysis. The results of the meta-analysis showed that the 3-year OS rate was slightly higher in the IMRT group than in the 3D-CRT group, without any statistical significance. The 3-year local control rate was significantly higher in the IMRT group than in the 3D-CRT group. No significant difference in the 3-year DFS rate was found between the IMRT and 3D-CRT groups. Grade 2–4 toxicities were similar between the IMRT and 3D-CRT groups.

**Conclusion:**

The findings suggested that IMRT might be superior to 3D-CRT in treating patients with gastric cancer in terms of local control rates without increasing toxicity.

**Electronic supplementary material:**

The online version of this article (10.1186/s13014-019-1294-0) contains supplementary material, which is available to authorized users.

## Background

Gastric cancer is a major cause of cancer-associated mortality worldwide [1]. Nearly half of the worldwide gastric cancer cases and deaths occur in China [1, 2]. As reported by the GLOBOCAN 2012, approximately one million new gastric cancer cases and more than 700,000 cancer-related deaths occurred globally in 2012 [3]. The treatment strategy for gastric cancer is still controversial [4]. Surgical resection is preferred for patients without advanced-stage cancer [4]. On the contrary, the benefits of surgical resection for patients with locally advanced gastric cancer are limited [5–8]. Perioperative strategies and adjuvant therapies, such as chemotherapy, radiotherapy, chemoradiotherapy, and targeted therapy, have been used in clinical settings for years and proved to be effective [4, 9–12].

Several clinical studies demonstrated that adjuvant therapies administered after surgical resection prolonged the survival of patients with locally advanced gastric cancer [4, 10, 11, 13–18]. Adjuvant therapy, such as radiotherapy, significantly increased the survival of patients with gastric cancer and reduced the risk of recurrence [15, 17]. However, the therapeutic benefits were accompanied by increased adverse events or toxicity [17, 19].

The three-dimensional conformal radiation (3D-CRT) and intensity-modulated radiation therapy (IMRT) are gradually implemented in clinical studies to reduce radiation-related toxicity without the loss of treatment effectiveness. A previous study showed significant grade 3 toxicity in patients with resected gastric or gastroesophageal junction cancers treated with 3D-planned CRT [20]. In recent years, several clinical trials have been performed to evaluate clinical outcomes and toxicity in patients with resected gastric cancer treated with IMRT versus 3D-CRT [21–28]. Minn et al. [29] compared the clinical efficacy and adverse events in patients with gastric cancer who received IMRT versus 3D-CRT. They did not find a significant difference between these two groups in terms of 2-year overall survival (OS) rate (*P* = 0.5). Whether IMRT was associated with reduced toxicity compared with 3D-CRT was explored by Liu et al. [26] who recruited 24 patients with stage IB–IIIB gastric cancer: 12 in the 3D-CRT group and 12 in the IMRT group. No significant differences in the OS and disease-free survival (DFS) rates were observed between 3D-CRT and IMRT, while similar toxicity was observed in these two groups. Due to the smaller sample size and nonsignificant differences observed in the aforementioned studies, this meta-analysis was performed to explore whether IMRT was more effective and safe compared with 3D-CRT in treating patients with gastric cancer.

## Method

### Search strategy

The electronic databases, including PubMed, Embase, and the Cochrane Library, were systematically searched using the following key words: gastric cancer, gastric carcinoma, stomach cancer, intensity-modulated radiotherapy, intensity-modulated radiation therapy, three-dimensional conformal radiation therapy, three-dimensional conformal radiotherapy, 3-dimensional conformal radiation therapy, IMRT, and 3D-CRT. The languages of the included studies were limited to English and/or Chinese. Relevant studies were manually retrieved if necessary. The details of the search strategy in PubMed are shown in Additional file 5: Data S1.

### Inclusion and exclusion criteria

#### Inclusion criteria

The study eligibility criteria were as follows: (1) patients diagnosed with gastric cancer; (2) two comparison groups, one group receiving IMRT and the other group receiving 3D-CRT; and (3) follow-up time: ≥6 months.

The report eligibility criteria were as follows: (1) outcomes including OS, DFS, and toxicity; (2) randomized controlled trials (RCTs) or observational study; (3) language limited to Chinese and/or English; (4) study sample size more than 15 cases; and (5) published studies and meeting abstracts.

#### Exclusion criteria

The exclusion criteria were as follows: (1) review, case report, abstracts, and lectures; (2) patients lacking precise clinical diagnosis; (3) incorrect data or incomplete data that could not be extracted from other relevant data; and (4) repeated published studies. The studies were selected according to the inclusion and exclusion criteria, and the data were extracted from the context of the studied reports.

#### Data extraction and quality assessment

Two independent investigators (FR and YZ) extracted the following essential information using a predesigned data extraction table that involved (1) general information, including the title, author, date of publication, and source of the study; (2) research characteristics, including general information regarding patients and interventions; and (3) survival rates, relapse rates, and toxicity in each group. The indicators of radiotherapy included PTV dose distribution, uniformity index (HI), average dose of normal liver (*D*_mean_), and average dose of kidneys (*D*_mean_). Discrepancies were discussed by these two reviewers. A third reviewer (YC) was consulted if any disagreement occurred. Inquiries to the original researchers were performed to collect additional or missing information.

The methodological quality of the studies was evaluated according to the quality evaluation criteria of RCTs detailed in the *Cochrane System Reviewer’s Manual 5*: (1) generation of random sequences; (2) allocation hiding; (3) blinding; (4) lost to follow-up and exiting; (5) selective reporting; and (6) other selective biases.

The study quality was assessed using the Newcastle–Ottawa scale (NOS). An NOS score ≥ 7 indicated high-quality studies [30, 31].

#### Statistical analysis

This meta-analysis was performed using the Stata 14.0 software (Stata Corp LLC). Appropriate statistical methods were used to analyze the differences in effectiveness and safety between the IMRT and 3D-CRT groups. The heterogeneity between the included studies was analyzed using the *I*^2^ test. If no heterogeneity was observed within studies (*P* > 0.1 and *I*^2^% < 50%), the fixed-effects model was applied for the analysis. Otherwise, the source of heterogeneity was detected using subgroup and meta-regression analyses. If statistical heterogeneity existed between studies without clinical heterogeneity, or if the difference was not clinically significant, the random-effects model was used for the analysis. The sensitivity analysis was applied to examine the stability of the meta-analysis results. Publication bias was assessed using funnel plots and Begg’s and Egger’s regression asymmetry tests [32, 33]. The risk ratio (RR) was used to present the dichotomous data, and the confidence interval (CI) was set to 95%. A difference with *P* value ≤0.05 was considered statistically significant.

## Results

### Results of literature research

**Fig. 1:**
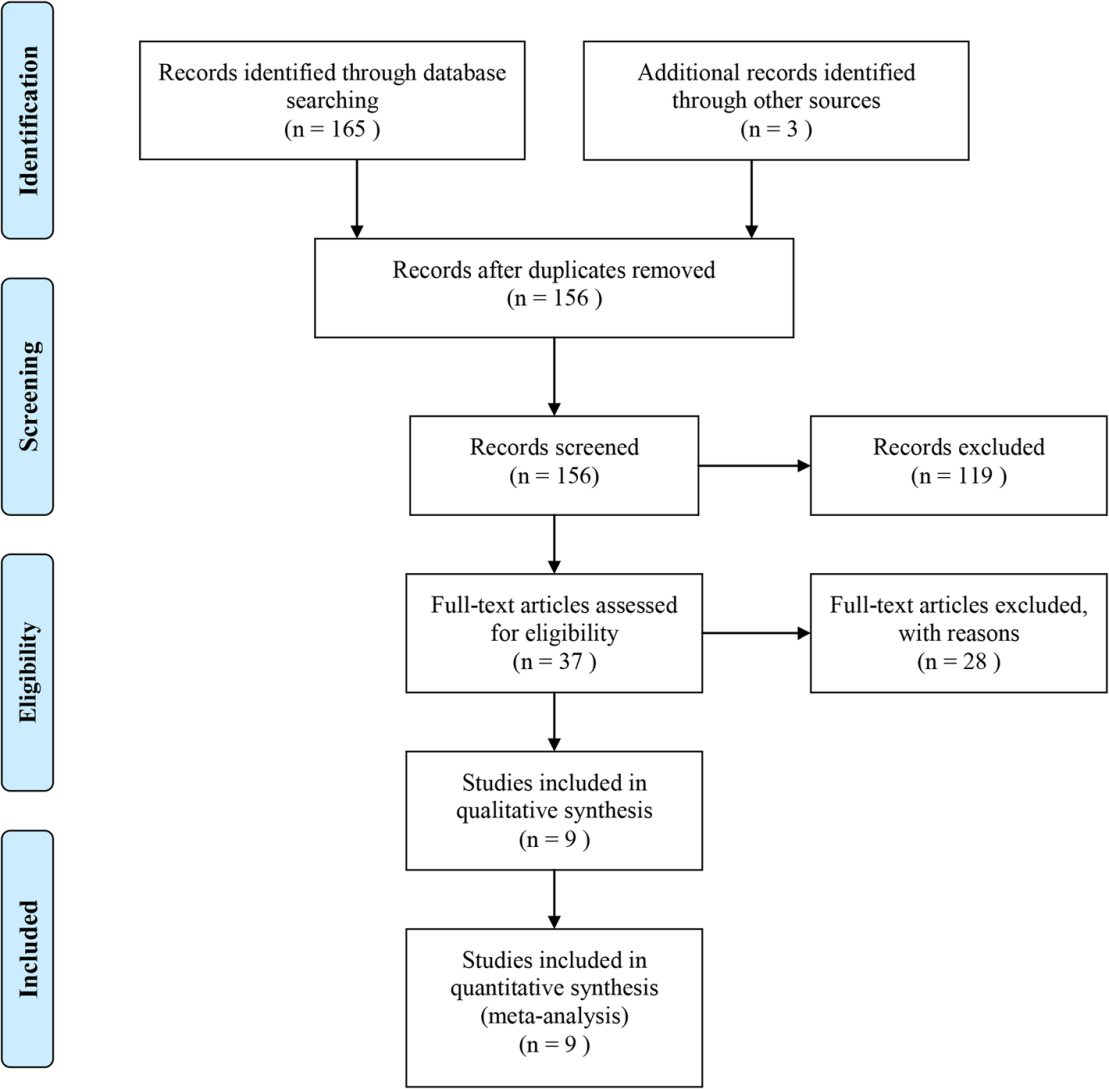
Flow chart for identifying eligible clinical studies

A total of 168 studies were identified after comprehensively searching relevant databases and other sources. Twelve studies were excluded due to duplication. After reviewing the titles and abstracts, 119 studies were excluded for multiple reasons. Further, 28 additional studies that did not meet the inclusion criteria were excluded after reviewing full texts. Finally, two RCTs, one prospective study, and 6 retrospective studies [12, 22, 23, 25, 26, 29, 34–36], involving 516 patients, were included in this meta-analysis. Of these, eight trials [12, 22, 23, 25, 26, 29, 34, 36] compared the efficacy and toxicity of IMRT versus 3D-CRT in patients with resected gastric cancer. Two RCTs [22, 25] evaluated the efficacy and toxicity of postoperative IMRT versus 3D-CRT. The basic characteristics of the included studies are shown in Table 1, and the trial selection process is presented in Fig. 1.

**Table 1:** Baseline characteristics of included studies

Author	Year	N	Sex(Male/Female)	Age	Disease status	Surgery	N in IMRT	N in 3D-CRT (Gy)	Dose of IMRT (Gy)	Dose of 3D-CRT	Fraction of IMRT	Fraction of 3D-CRT	Chemotherapy	Type	Stage (AJCC)
Xin Wang [22]	2016	100	82/28	55 (23–73)	stage IB-IIIC gastric cancer	curative resection with D2 lymph node dissection	42	68	50.4	50.4	28	28	5-FU+ CDDP	RCT	IB-IIIC
A. Yuriko Minn [29]	2010	57	38/19	58 (29–83)	gastric or gastroesophageal junction cancer	Esophagogastrectomy, total or subtotal gastrectomy	31	26	45	45	25	25	5-FU+ CDDP	RS	I-III
Gene-Fu F. Liu [26]	2014	24	18/6	56–64	stage IB-IIIB gastric cancer	Esophagogastrectomy, total or subtotal gastrectomy	12	12	50.4	45	25	25	5-FU	RS	IB-IIIC
Boda-Heggemann J [12]	2009	60	37/23	35–75	gastric or gastroesophageal junction cancer	D2 resection	33	27	45	45	25	25	5-FU+ CDDP	RS	I-IV
Boda-Heggemann J [34]	2013	65	38/27	35–76	gastric or gastroesophageal junction cancer	total or subtotal gastrectomy	38	27	45	45	25	25	5-FU+ CDDP	RS	I-IV
Chopra S [23]	2015	51	35/16	54 (25–74)	gastric or, proximal gastric and gastro-esophageal junction cancer	total or subtotal gastrectomy	26	25	45	45	25	25	Capecitabine	RS	I-III
Goody, R. B. [35]	2016	55	30/25	54 (28–77)	completely resected gastric cancer	total or subtotal gastrectomy	NA	NA	45	45	25	25	cisplatin	PS	I-III
Fang, L. [25]	2015	75	54/21	47 (31–58)	stage III-IV gastric cancer	curative resection	25	25	45	45	NA	NA	None	RCT	III-IV
Deng, Q. H. [36]	2011	29	23/6	55 (29–72)	stage III-IV gastric cancer	total or subtotal gastrectomy	29	29	45	45	25	25	None	RS	III-IV (UICC)

Abbreviation: *N* number, *IMRT* intensity modulated radiation therapy, *3D-CRT* three-dimensional conformal radiation treatment, *RCT* randomized controlled trial, *5-Fu* 5-fluorouracil, *NA* not available, *RS* retrospective study, *PS* prospective study, *AJCC* American Joint Committee on Cancer, *UICC* UnionInternationale Against Cancer

### General information and characteristics of the included studies

#### Patients and interventions

A total of 516 patients with gastric cancer participated in the eligible clinical trials. The IMRT group and the 3D-CRT group involved 236 and 239 patients, respectively. The age of all included patients ranged from 23 to 83 years. Most of the patients underwent surgical resection of gastric cancer. This radiotherapy was performed by clinical radiation oncologists, physicists, and technicians. The definitions and details of radiation fields were different among the included studies. A variety of radiation doses of IMRT and 3D-CRT were used in the studies for treating gastric cancer. The duration of radiation ranged from 5 to 6 weeks. Traditional supportive treatments and/or adjuvant chemotherapy were used for patients during radiotherapy in some studies. The baseline characteristics of the eligible studies are listed in Table 1.

#### Quality assessment of included studies

The overall methodological quality of all included studies was high. Two RCTs [22, 25] used random allocation hiding, and all reported cases lost to the follow-up. The quality assessment of the included studies is shown in Additional file 6: Table S1 and Additional file 7: Table S2.

### Effectiveness of interventions

#### Overall survival

**Fig. 2:**
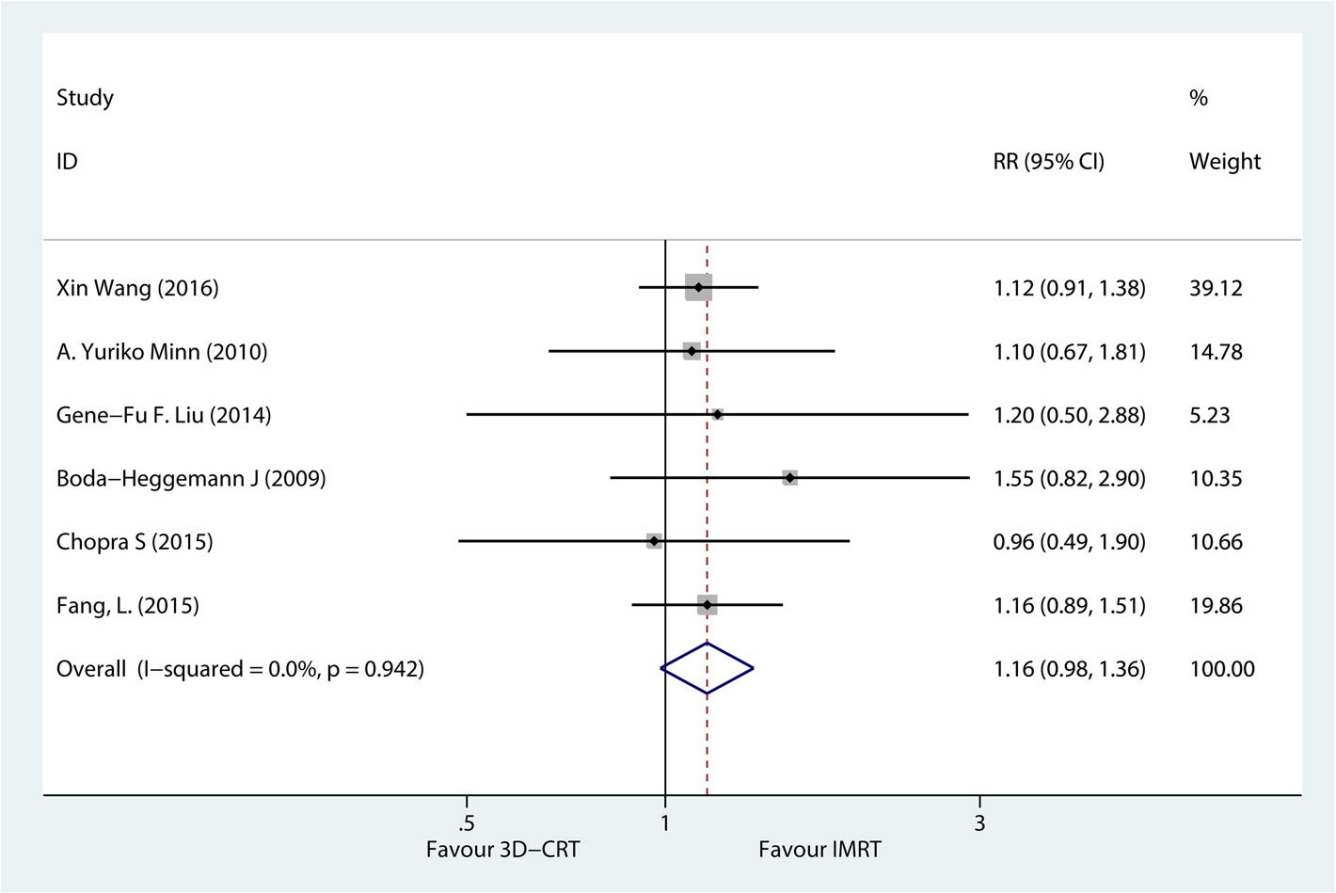
Comprehensive analysis of the impact of IMRT versus 3D-CRT on the 3-year overall survival rate in patients with resected gastric cancer

Six studies, including 352 patients, compared the OS rate of IMRT versus 3D-CRT in patients with gastric cancer. The heterogeneity test results were *P* = 0.942 and *I*^2^ = 0%, indicating a low risk of heterogeneity; the fixed-effects model was then used. The forest plots of the meta-analysis showed that patients with IMRT had a slightly better 3-year OS rate with an RR of 1.16, compared with patients with 3D-CRT (95% CI, 0.98–1.36) (Fig. 2) despite no statistical significance.

Two studies [12, 29] investigated the 2-year OS rate, while one study [34] reported the 5-year OS rate in patients with gastric cancer. The heterogeneity test results were *P* = 0.42 and *I*^2^% = 0%, showing no heterogeneity within studies. The results of the meta-analysis indicated that IMRT was associated with a significantly better 2-year OS rate with a pooled RR of 2.49 (95% CI, 1.18–5.25; *P* = 0.02) compared with 3D-CRT. The descriptive analysis of the 5-year OS rate was used due to the lack of enough data for combined analysis. Boda-Heggemann et al. [34] reported that the 5-year OS rate in the IMRT and 3D-CRT groups was 0.47 (18/38) and 0.26 (7/27), respectively.

#### Disease-free survival

**Fig. 3:**
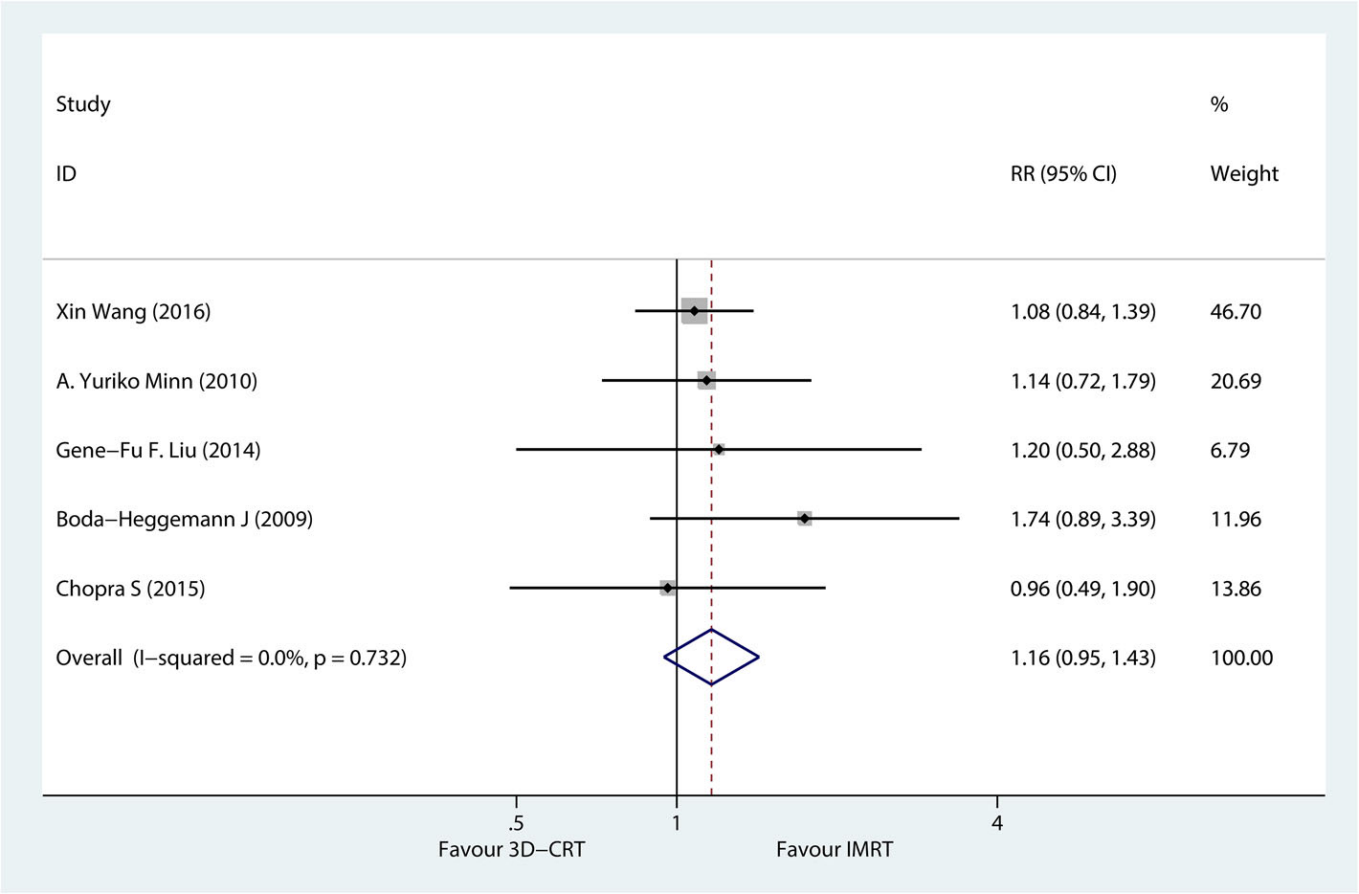
A fixed-effects meta-analysis of the impact of postoperative IMRT versus 3D-CRT on the 3-year disease-free survival rate in patients with gastric cancer

As shown in Fig. 3, 5 studies, including 302 patients with gastric cancer, investigated the DFS rate after IMRT and 3DCRT. The 3-year DFS rate was similar in patients receiving IMRT and 3D-CRT, with no heterogeneity (*P* = 0.732; *I*^2^% = 0%). The administration of IMRT was not associated with an improvement in the DFS rate (RR = 1.16; 95% CI, 0.95–1.43; *P* > 0.05). Boda-Heggemann et al. [34] reported that the 5-year DFS rate in the IMRT and 3D-CRT groups was 0.44 (17/38) and 0.22 (6/27), respectively.

#### Loco-regional relapse rate

**Fig. 4:**
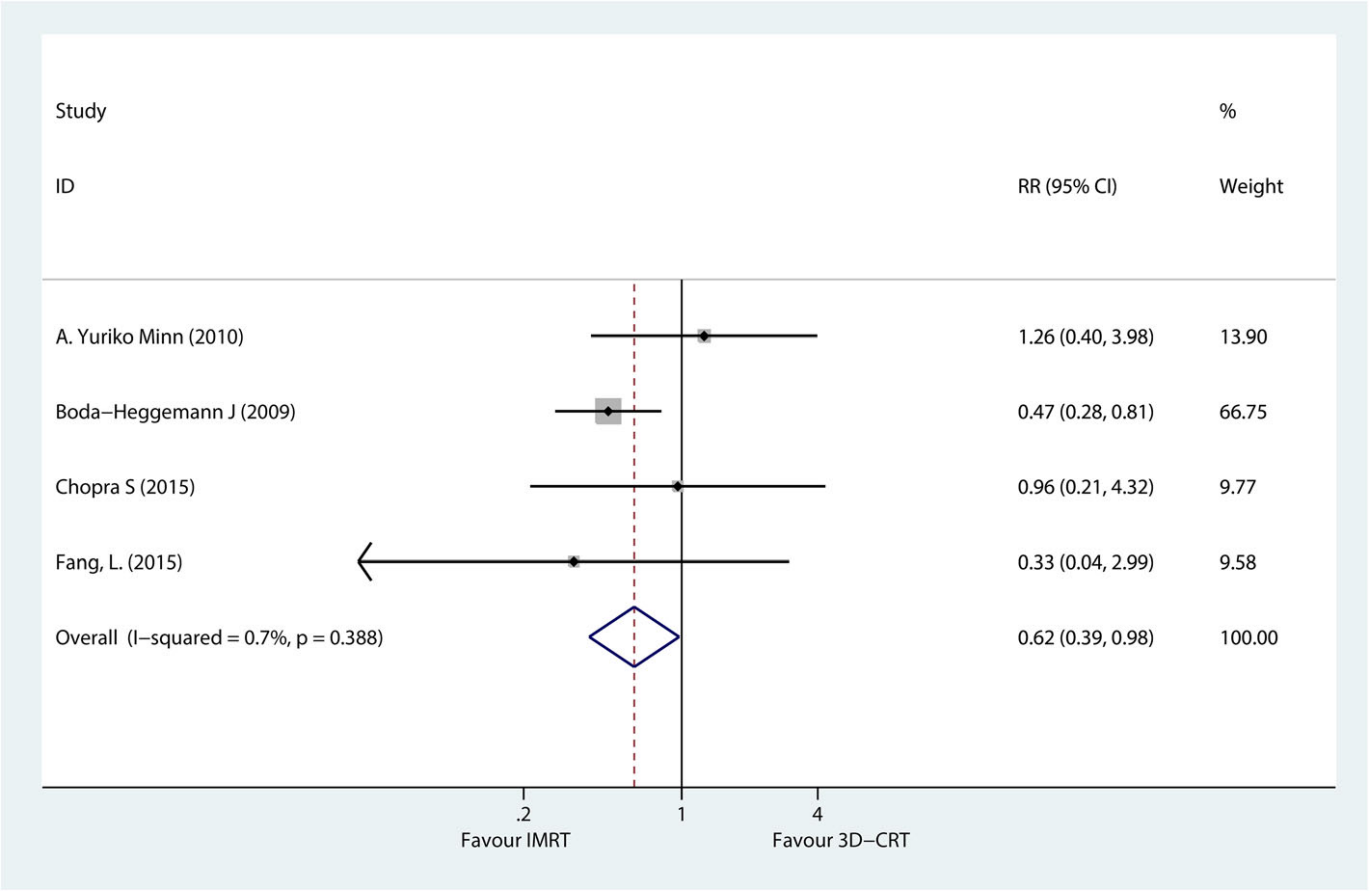
A fixed-effects meta-analysis of the impact of postoperative IMRT versus 3D-CRT on the 3-year loco-regional recurrence in patients with gastric cancer

Four studies, including 218 patients, compared the loco-regional recurrence (LRR) rate in patients with gastric cancer after receiving IMRT versus 3D-CRT. The meta-analysis results showed statistically significant differences between the groups (RR = 0.62; 95% CI, 0.39–0.98; *P* < 0.05), indicating a decreased risk of 3-year LRR in the IMRT group compared with the 3D-CRT group (Fig. 4).

#### Quality of life

Goody et al. [35] reported the changes in the quality of life of patients with gastric cancer treated with IMRT versus 3D-CRT. The quality-of-life compliance ranged from 93% at baseline to 70% after 4 weeks of the treatment.

#### Toxicity

**Fig. 5:**
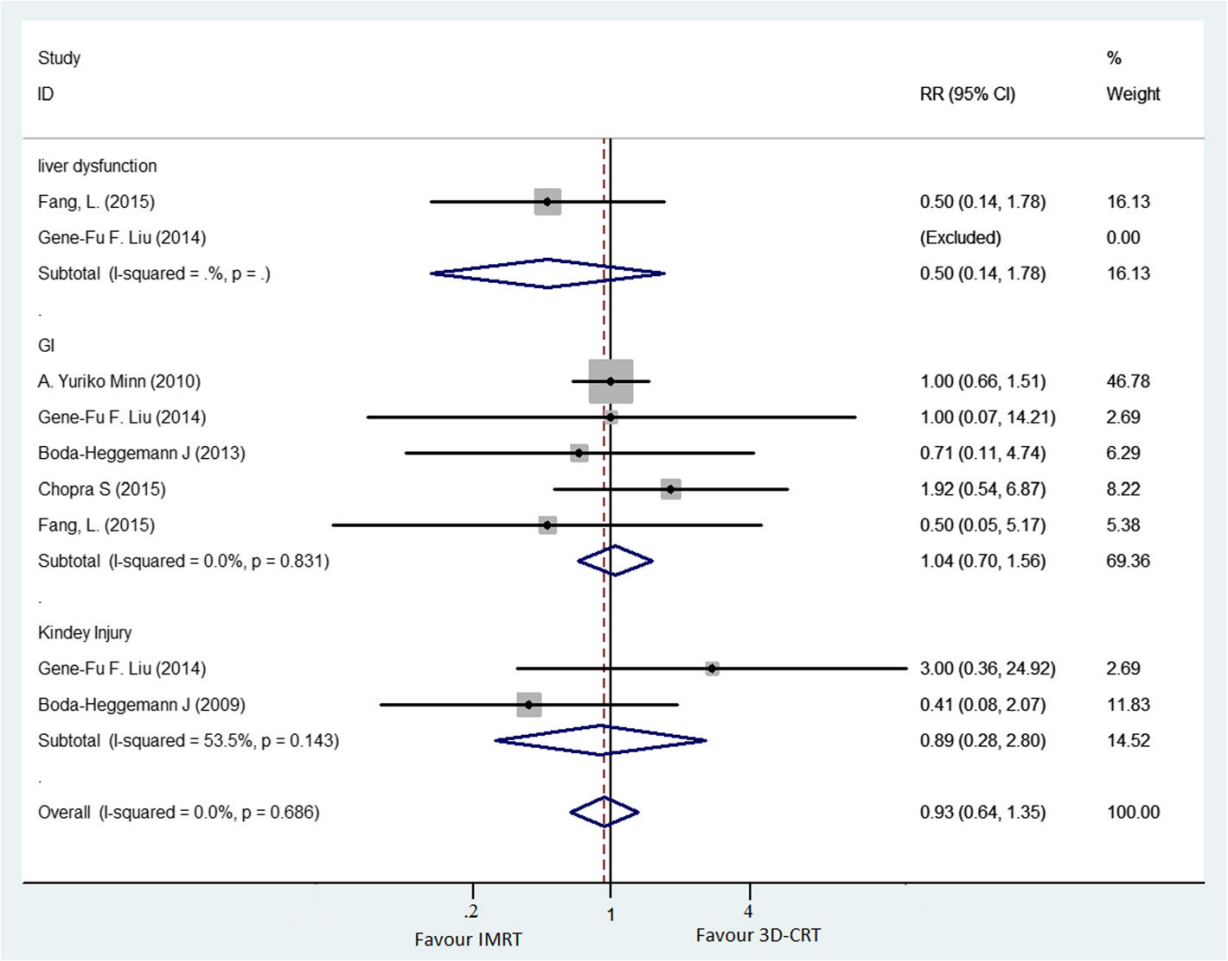
A fixed-effects meta-analysis of the impact of IMRT versus 3D-CRT on toxicity. Trials were grouped by study with respect to toxicity

An overview of the toxicities reported in included trials is presented in Fig. 5. The validated definition of radiation-related toxicities was based mainly on the Radiation Therapy Oncology Group or Common Toxicity Criteria scales in these selected studies. Three studies [22, 35, 36] did not show detailed toxicity data for analysis.

Liver dysfunction, gastrointestinal (GI) toxicity, and kidney toxicity were reported in two studies, five studies, and two studies, respectively (Fig. 5). No significant differences in liver dysfunction, GI toxicity, and kidney toxicity were found in patients receiving postoperative IMRT compared with 3D-CRT. The RR for liver dysfunction, GI toxicity, and kidney toxicity was 0.5 (95% CI, 0.14–1.78), 1.04 (95% CI, 0.70–1.56), and 0.89 (95% CI, 0.28–2.80), respectively (Fig. 5).

#### Sensitivity analysis

A sensitivity analysis of all the four meta-analyzed outcomes (OS, DFS, LRR, and toxicity) was performed. Except for LRR, all the pooled results of the other three outcomes (OS, DFS, and toxicity) did not change significantly (Additional file 1: Figure S1, Additional file 2: Figure S2, Additional file 3: Figure S3, Additional file 4: Figure S4). The pooled LRR result changed after excluding the study by Boda-Heggemann et al. [34], indicating that this study weighted largely (66.75%).

#### Publication bias

The funnel plots and Egger’s and Begg’s tests were used to detect any publication bias. No significant evidence of publication bias in the meta-analysis of OS rate was found (Egger’s test: *P* = 0.684; Begg’s test: *P* = 0.707).

## Discussion

This meta-analysis examined the impact of IMRT versus 3D-CRT in patients with resectable gastric cancer by quantitatively summarizing the findings from nine different trials. The results showed that the use of IMRT was associated with a nearly 40% reduction in the risk of disease relapse and a 16% increase in the OS rate. Moreover, the risk of radiation-related toxicities was not increased in IMRT compared with 3D-CRT. Taken together, IMRT seemed to be a promising alternative in this clinical setting.

A meta-analysis [16] assessed the impact of radiotherapy on both 3- and 5-year survival [overall survival (OS) and disease-free survival (DFS)] rates in patients with resectable gastric cancer by including 14 RCTs. The pooled result showed that the addition of radiotherapy after surgery improved the 3- (RR, 1.18; 95% CI, 1.01–1.38) and 5-year OS and DFS rates (RR, 1.38; 95% CI, 1.18–1.61). IMRT was widely used in recent studies, demonstrating promising efficacy and less toxicity. Therefore, comparing the efficacy and toxicity of IMRT with those of 3D-CRT in patients with gastric cancer was necessary. The findings of this meta-analysis indicated a significant improvement in patient’s outcomes after IMRT compared with 3D-CRT. Notably, IMRT significantly reduced the risk of loco-regional relapse, indicating its high clinical efficacy and potential as first-line adjuvant treatment for locally advanced or high-risk gastric cancer.

Reducing radiation toxicity and improving treatment compliance and quality of life of patients are serious issues in clinical practice. This meta-analysis showed that patients in the IMRT group did not experience increased radiation-related side effects compared with those in the 3D-CRT group. Recent studies showed that IMRT had a lower incidence of toxicity, especially grade 3 and 4 toxicities, compared with 3D-CRT. Murthy et al. [37] showed that IMRT was more advantageous than 3D-CRT in terms of dose coverage and conformity. Ringash et al. [38] also reported that the conformity and uniformity of IMRT were better than those of 3D-CRT; it was better in reducing the dose of liver radiation, thus decreasing liver toxicity. In addition, Wei Gang et al. [39] also showed that the radiation field distribution, homogeneity, and conformity of IMRT were superior to those of 3D-CRT in reducing the normal tissue radiation dose. Wieland et al. [40] showed that the radiation dose of the kidney and liver was lower in the IMRT group than in the 3D-CRT group. The results were consistent with those of previous IMRT studies, which paved the way for IMRT as the stand-alone radiotherapy treatment for gastric cancer.

Although a strict retrieving and analysis strategy was used for a comprehensive meta-analysis, some limitations should be highlighted. First, the included studies had uneven quality and a limited number of participants; some of them were retrospective studies, increasing the risk of selective reporting bias. Second, the included studies were limited to Chinese and English databases, leading to language bias. Third, the heterogeneity of IMRT or 3D-CRT in different studies led to clinical heterogeneity and reduced the statistical power. Fourth, the survival data were not provided by different age and staging of patients with gastric cancer, particularly the lymph node status after resection. Therefore, it was not appropriate to analyze the impact of IMRT versus 3D-CRT on patient survival by age and tumor staging. Finally, the detail and radiation fields of radiotherapy varied among studies, leading to different toxicities and efficacy. The radiation dose of IMRT was higher than that of 3D-CRT in the study by Liu et al. [26], but severe toxicity was similar for the two. The present meta-analysis also showed similar toxicity for IMRT and 3D-CRT, which was consistent with previous findings. Despite the aforementioned limitations, the findings of this meta-analysis might guide adjuvant therapies for resected gastric cancer.

## Conclusions

In summary, this meta-analysis showed that IMRT was associated with a slight increase in the 3-year OS rate and a significant increase in the local control rate, without affecting the DFS rate or increasing the clinical toxicity rate, compared with 3D-CRT. Further studies, such as more rigorous, high-quality RCTs, are required to validate the effectiveness of IMRT in treating gastric cancer. Moreover, a dose–response curve for the radiation dose and potential injuries at specific sites should be explored.

## Additional files

Additional file 1: **Figure S1.** Overall survival sensitivity. (TIF 383 kb)

Additional file 2: **Figure S2.** Disease-free survival sensitivity. (TIF 353 kb)

Additional file 3: **Figure S3.** Loco-regional recurrence sensitivity. (TIF 315 kb)

Additional file 4: **Figure S4.** Toxicity sensitivity. (TIF 468 kb)

Additional file 5: Data S1. Search strategy in PubMed. (DOCX 17 kb)

Additional file 6: **Table S1.** Methodological quality of randomized controlled trials assessed using the Cochrane risk-of-bias tool. (DOC 30 kb)

Additional file 7: **Table S2.** Methodological quality of cohorts assessed using the Newcastle–Ottawa scale. (DOCX 18 kb)

## Abbreviations

3D-CRT: Three-dimensional conformal radiation
CI: Confidence interval
GI: Gastrointestinal
IMRT: Intensity-modulated radiation therapy
NOS: Newcastle–Ottawa scale
RCTs: Randomized controlled trials
RR: Risk ratio
