# Supplementary figures and images for: Efficacy and safety of intensity-modulated radiation therapy versus three-dimensional conformal radiation treatment for patients with gastric cancer: a systematic review and meta-analysis
Source: Radiat Oncol. 2019 May 22;14:84. doi: 10.1186/s13014-019-1294-0 (PMC6532249; doi:10.1186/s13014-019-1294-0)

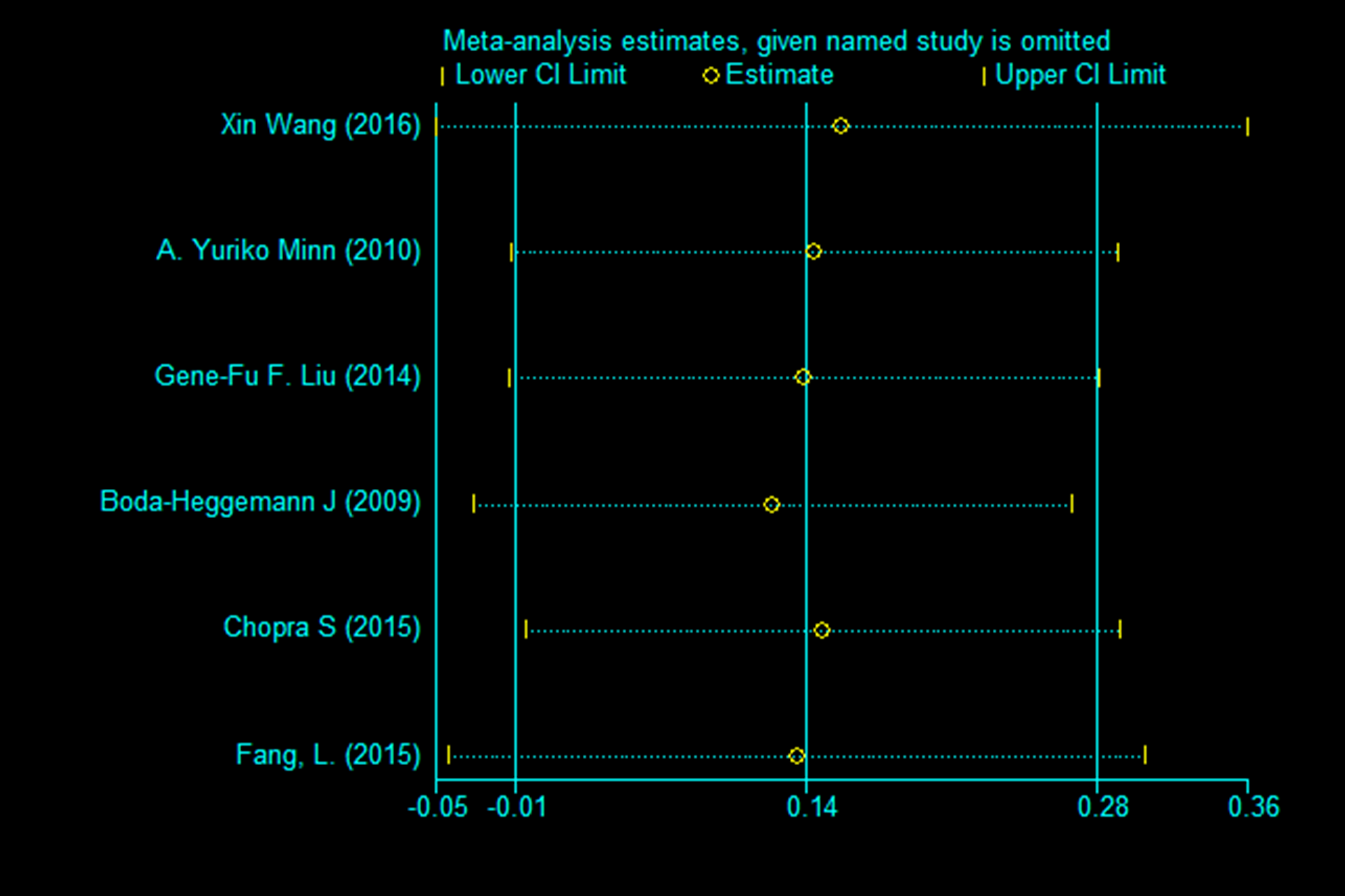

Supplement: Supplementary file 1 — Figure S1. Overall survival sensitivity. (TIF 383 kb) [file 13014_2019_1294_MOESM1_ESM.tif]

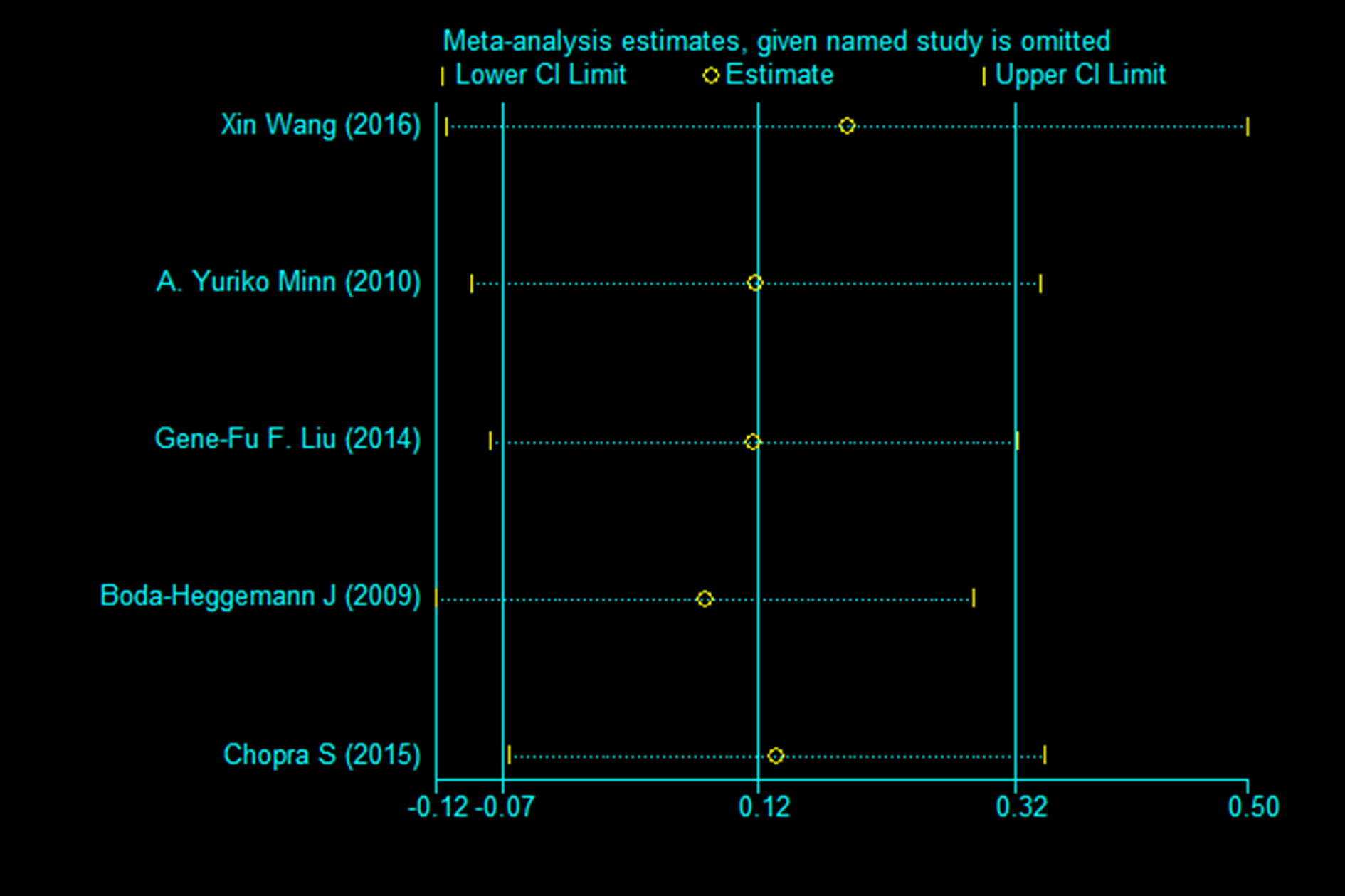

Supplement: Supplementary file 2 — Figure S2. Disease-free survival sensitivity. (TIF 353 kb) [file 13014_2019_1294_MOESM2_ESM.tif]

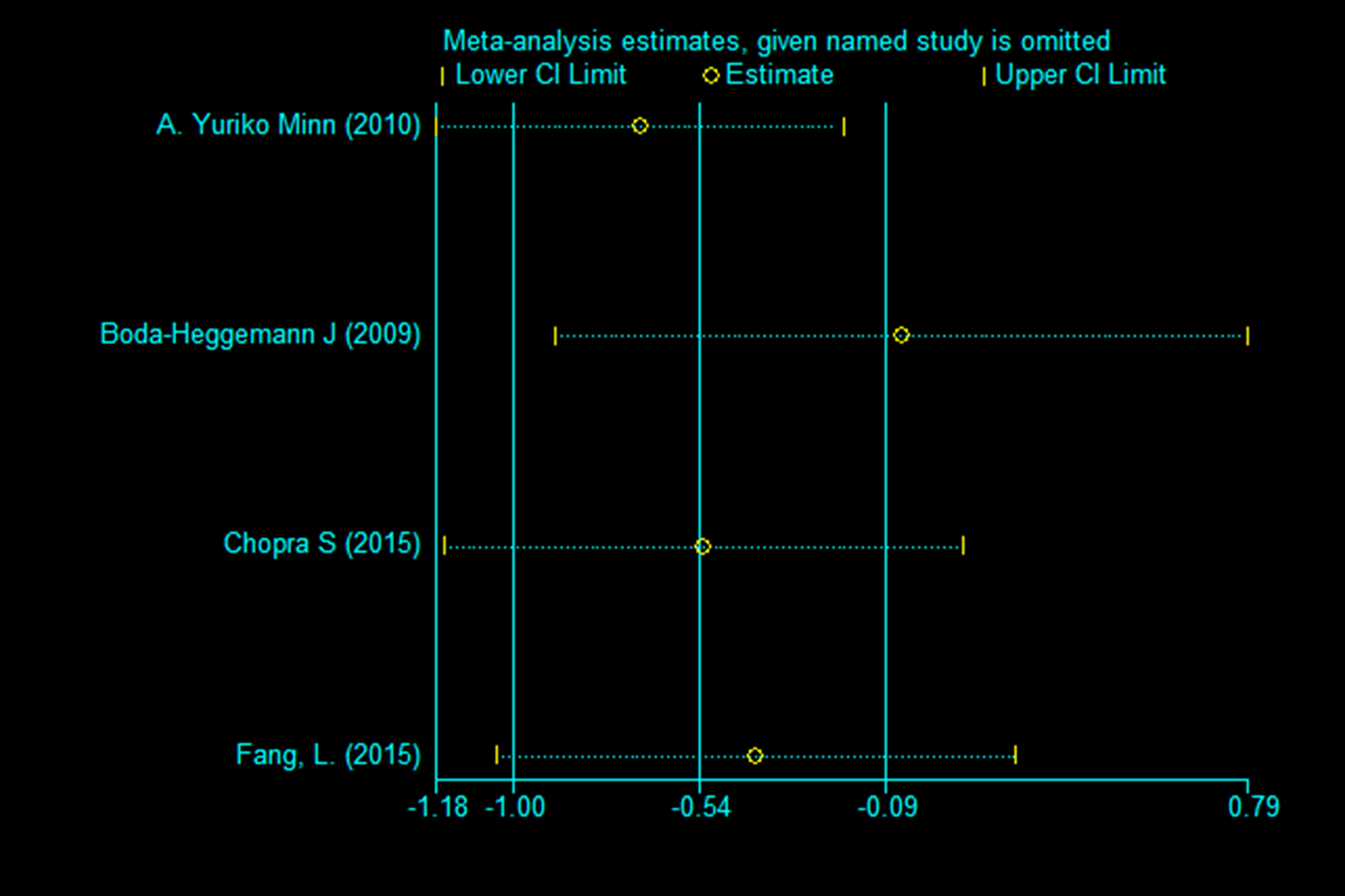

Supplement: Supplementary file 3 — Figure S3. Loco-regional recurrence sensitivity. (TIF 315 kb) [file 13014_2019_1294_MOESM3_ESM.tif]

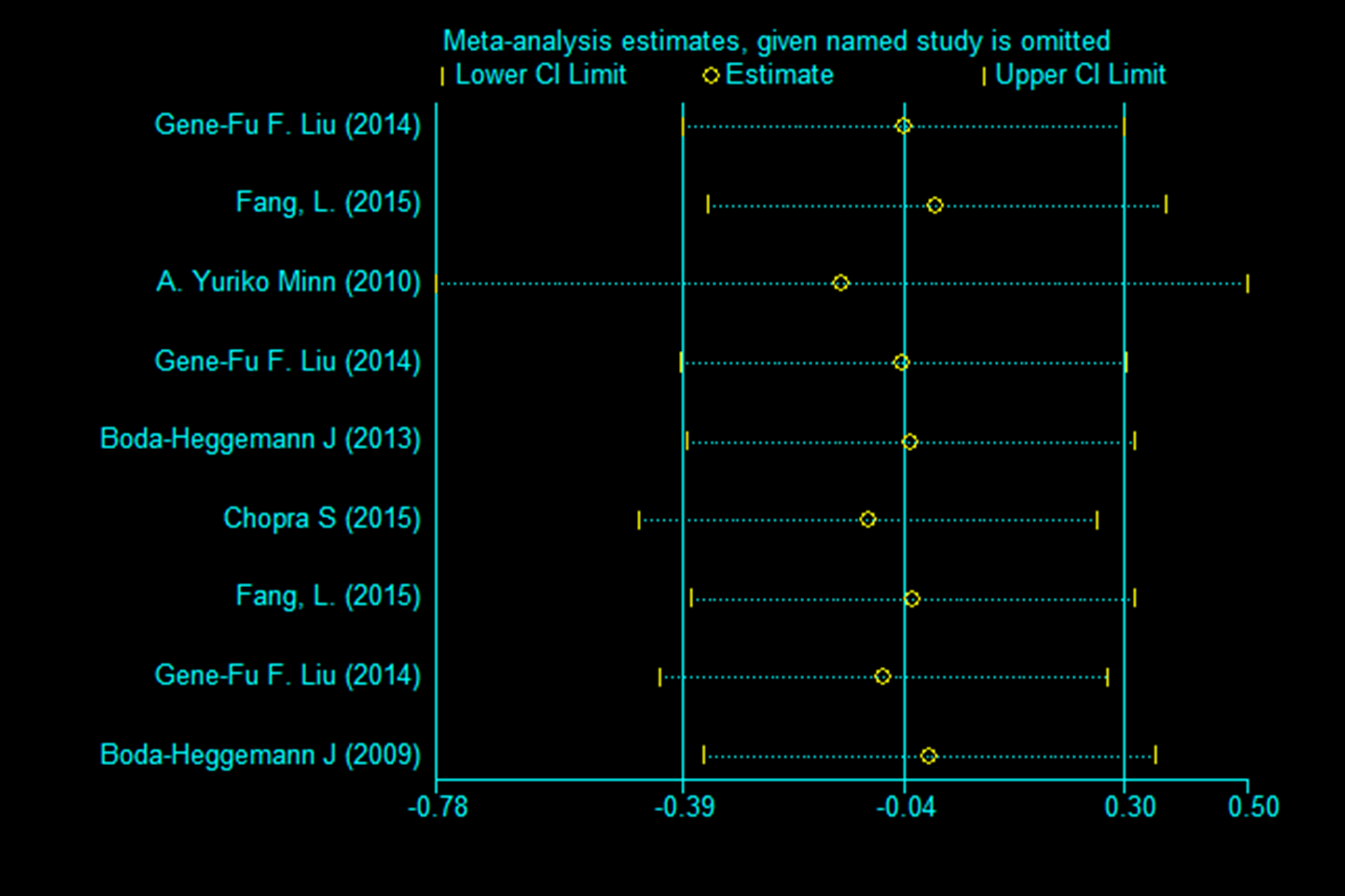

Supplement: Supplementary file 4 — Figure S4. Toxicity sensitivity. (TIF 468 kb) [file 13014_2019_1294_MOESM4_ESM.tif]
