# Supplementary material for: Efficacy and safety of intensity-modulated radiation therapy versus three-dimensional conformal radiation treatment for patients with gastric cancer: a systematic review and meta-analysis
Source: Radiat Oncol. 2019 May 22;14:84. doi: 10.1186/s13014-019-1294-0 (PMC6532249; doi:10.1186/s13014-019-1294-0)
Supplement: Supplementary file 5 — Data S1. Search strategy in PubMed. (DOCX 17 kb) [file 13014_2019_1294_MOESM5_ESM.docx]

Supplemental Data 1. The search strategy in PubMed.

#1: Search "Stomach Neoplasms"[Mesh]

#2: Search ((((((Cancer of the Stomach[Title/Abstract]) OR Neoplasm, Stomach[Title/Abstract]) OR Stomach Neoplasm[Title/Abstract]) OR Gastric Neoplasms[Title/Abstract]) OR Gastric Cancer of Stomach[Title/Abstract]) OR Stomach Cancers[Title/Abstract]) OR Gastric Cancer[Title/Abstract]

#3: #1 OR #2

#4: Search ((three-dimensional conformal radiation[Title/Abstract]) OR conformal radiation[Title/Abstract]) OR 3D-CRT[Title/Abstract]

#5: Search "Radiotherapy, Intensity-Modulated"[Mesh]

#6: Search (((((Arc Therapies, Volumetric-Modulated[Title/Abstract]) OR Intensity-Modulated Radiotherapies[Title/Abstract]) OR Intensity-Modulated Radiotherapy[Title/Abstract]) OR Radiotherapies, Intensity-Modulated[Title/Abstract]) OR Radiotherapy, Intensity Modulated[Title/Abstract]) OR Volumetric-Modulated Arc Therapy[Title/Abstract]

#7: #5 OR #6

#8: #3 AND #4 AND #7
