# Supplementary material for: Efficacy and safety of intensity-modulated radiation therapy versus three-dimensional conformal radiation treatment for patients with gastric cancer: a systematic review and meta-analysis
Source: Radiat Oncol. 2019 May 22;14:84. doi: 10.1186/s13014-019-1294-0 (PMC6532249; doi:10.1186/s13014-019-1294-0)
Supplement: Supplementary file 6 — Table S1. Methodological quality of randomized controlled trials assessed using the Cochrane risk-of-bias tool. (DOC 30 kb) [file 13014_2019_1294_MOESM6_ESM.doc]

Supplemental table 1 The methodological quality of RCTs by the Cochrane risk qualified tool

| Author | 1) generation of random sequences | 2) allocation hiding | 3) blinding | 4) lost to follow-up and exit | 5) selective reporting | 6) other selective bias |
| --- | --- | --- | --- | --- | --- | --- |
| Xin Wang 2016 | ** | ** | ** | ** | ** | * |
| Fang L 2015 | ** | ** | ** | ** | ** | * |

**: low risk, *: unclear, 0: high risk
