# Supplementary material for: Efficacy and safety of intensity-modulated radiation therapy versus three-dimensional conformal radiation treatment for patients with gastric cancer: a systematic review and meta-analysis
Source: Radiat Oncol. 2019 May 22;14:84. doi: 10.1186/s13014-019-1294-0 (PMC6532249; doi:10.1186/s13014-019-1294-0)
Supplement: Supplementary file 7 — Table S2. Methodological quality of cohorts assessed using the Newcastle–Ottawa scale. (DOCX 18 kb) [file 13014_2019_1294_MOESM7_ESM.docx]

Supplemental table 2 The methodological quality of cohorts by NOS

|  | **Selection** | | | | **Comparability** | **Outcome** | | | **total** |
| --- | --- | --- | --- | --- | --- | --- | --- | --- | --- |
| Author | 1) Representativeness of the exposed cohort | 2) Selection of the non-exposed cohort | 3) Ascertainment of exposure | 4) Demonstration that outcome of interest was not present at start of study | 1) Comparability of cohorts on the basis of the design or analysis | 1) Assessment of outcome | 2) Was follow-up long enough for outcomes to occur | 3) Adequacy of follow up of cohorts |  |
| Gene-Fu F. Liu 2014 | * | * | * | * | ** | * | * | * | 9 |
| A. Yuriko Minn 2010 | * | * | * | * | ** | * | * | * | 9 |
| Boda-Heggemann J 2009 | * | * | * | * | * | * | * | * | 8 |
| Boda-Heggemann J 2013 | * | * | * | * | * | * | * | * | 8 |
| Chopra S 2015 | * | * | * | * | ** | * | * | * | 9 |
| Goody R. B 2016 | * | * | * | * | ** | * | * | * | 9 |
| Deng Q. H 2011 | * | * | * | * | ** | * | 0 | * | 8 |
